# REACTIONS TO AMYLOID PET SCAN RESULTS AND LEVELS OF ANXIETY AND DEPRESSION AMONG CARE PARTNERS: CARE IDEAS STUDY

**DOI:** 10.1093/geroni/igz038.494

**Published:** 2019-11-08

**Authors:** Emmanuelle Belanger, Jessica D’Silva, Courtney H Van Houtven, Megan Shepherd-Banigan, Valerie Smith, Terrie Wetle

**Affiliations:** 1 Brown University, School of Public Health, Providence, Rhode Island, United States; 2 Durham VA HSR&D, Durham, North Carolina, United States; 3 Duke University, Durham, North Carolina, United States

## Abstract

Few studies have examined caregiver reactions to their loved ones receiving the results of an Amyloid PET scan which can be indicative of Alzheimer’s disease. Therefore, we examine: 1) What are care partner’s reactions to their loved one receiving negative or positive amyloid PET scan results?, and 2) To what extent are scan results and diagnostic category (dementia vs. mild cognitive impairment) associated with care partner depressive symptoms (PHQ-2) and anxiety (STAI-6)? Using data from 1,799 care partners in the CARE IDEAS study, we applied a sequential mixed-methods design and explored the reactions of 192 care partners who answered open-ended interview questions after learning about the Amyloid PET scan results. We first conducted qualitative content analysis of transcripts from open-ended questions to explore caregivers’ emotional responses after their loved one received an Amyloid PET scan result. The qualitative data suggest that when the scan results fit care partner’s expectations, i.e. positive scan when the patient has dementia and negative scan when the patient has mild impairment, care partners report satisfaction with this information and relief, rather than shock and frustration. Adjusted logistic regression models of survey responses support this finding; having dementia and a positive scan both increased the likelihood of care partners having high levels of anxiety, and a significant interaction indicated that a positive scan was associated with high anxiety among care partners of patients with mild cognitive impairment but not dementia. Only lower education and higher impairment in everyday cognitive function were associated with high depressive symptoms.

